# Managing systemic venous occlusions in children

**DOI:** 10.1186/s42155-020-00150-1

**Published:** 2020-11-15

**Authors:** Anne E. Gill, Giridhar M. Shivaram

**Affiliations:** 1 Department of Radiology & Imaging Sciences, Division of Interventional Radiology and Image-Guided Medicine, Emory University School of Medicine, 1405 Clifton Rd NE, Atlanta, GA 30322 USA; 2 Department of Radiology, Division of Interventional Radiology, University of Washington School of Medicine, Seattle, USA

**Keywords:** Systemic venous occlusion, Endovascular venous reconstruction, Pediatrics, Superior vena cava, Inferior vena cava, Endovascular stenting, Thromboembolism

## Abstract

Pediatric venous disease is increasing in incidence in both inpatient and outpatient populations. The widespread use of central venous access devices as well as the rising incidence of thromboembolic events in pediatrics is leading to more systemic venous occlusions in both the central and peripheral veins. This review focuses on the etiology, presentation, workup, and general technical considerations of recanalization as well as procedural complications related to pediatric systemic venous occlusive disease. The potential role for pediatric interventional radiology guided treatments will be discussed in detail.

## Introduction

Systemic venous occlusions (SVO) are an important subset of pediatric vascular disease. These can be congenital or acquired, the latter is often associated with long-term central venous catheters. Occlusions can be partial or complete and can result in fewer options for central venous access, deep vein thrombosis (DVT) and pulmonary embolism, and peripheral symptoms such as arm or leg swelling. The ability to successfully recanalize pediatric SVOs and perform thrombectomy when appropriate is paramount.

Barriers to successful management of SVOs in children include unfamiliarity with congenitally altered venous anatomy or the small patient, unfamiliarity with pediatric dosing of anticoagulants and thrombolytic drugs, unavailability of suitable equipment, and a perceived sense of heightened risk for recanalization procedures in children.

In this review, we discuss the etiology and presentation of SVOs in children, periprocedural medical management, general technical considerations of recanalization, strategies for addressing both acute and chronic or congenital occlusions, and procedural complications.

## Background

The overall prevalence of SVO in children is difficult to ascertain because most children with these types of occlusions are asymptomatic (Andrew et al. 1994; Frazer and Ing 2009). Menéndez et al. published their work following 265 children who underwent peripherally inserted central catheter placement for various indications and found that ~ 8% had line-associated thromboses, the majority of which were asymptomatic (Menéndez et al. 2016). SVO can be subdivided into congenital and acquired etiologies (Table 1), and in some cases there can be superimposition of acquired venous obstruction upon congenitally narrowed veins. Congenital SVOs in children are secondary to developmental hypoplasia or aplasia of major conducting veins (e.g. lower extremity deep vein anomalies seen in Klippel-Trenaunay syndrome) or in utero thrombosis of these conducting pathways. A variety of syndromes can result in inferior vena cava (IVC) atresia or obstruction and the development of aberrant conducting pathways (Bass et al. 2000). When infants present with SVO, a careful history should be obtained to determine whether the occlusion was congenital, the result of central venous catheterization, or other insults.

**Table 1:** Etiologies of Systemic Venous Occlusion (SVO)

Congenital SVO	Acquired SVO
Developmental hypoplasia of systemic veins (i.e. Klippel Trenaunay syndrome hypoplastic deep veins in affected extremity)	Catheter-associated obstruction
Developmental aplasia of systemic veins (i.e. IVC atresia)	Hypercoagability/thrombophilia
In-utero thrombosis	Mechanical obstruction (i.e. May Thurner syndrome)
	Trauma

Acquired etiologies of SVO include catheter-associated obstruction, most commonly in the iliofemoral/IVC or innominate/superior vena cava (SVC) distributions, deep vein thrombosis from hypercoagulability (e.g. Factor V Leiden deficiency) or mechanical obstruction (e.g. May-Thurner syndrome), and trauma. Distinguishing between congenital and acquired SVO is sometimes a diagnostic challenge especially if children do not present with symptoms of venous obstruction until later in life. However, making this distinction is critical because recanalization of congenital SVO is often impossible or may require aggressive techniques such as stent construction of a non-anatomic vascular channel. On the other hand, if an obstructed pathway is still present, even if diminutive from chronic obstruction and scarring, anatomic recanalization may be feasible. In the authors’ experience, reconstruction of native vasculature is associated with greater long-term patency compared to neovessel construction.

**Fig. 1:**
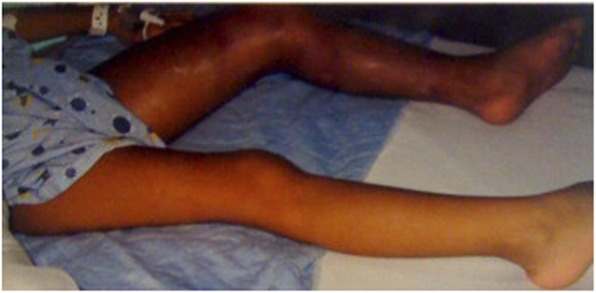
5-year-old female with history of May-Thurner syndrome presenting with acute left lower extremity DVT and skin changes characteristic of phlegmasia cerulea dolens. [Image adapted from Kuo I, Smith J, Abou-Zamzam AM Jr. A multimodal therapeutic approach to phlegmasia cerulea dolens in a pediatric patient. J Vasc Surg. 2011. Jan;53(1):212–5. doi: 10.1016/j.jvs.2010.07.067. Epub 2010 Sep 26. PubMed PMID: 20875715]

Clinical presentation of SVO in children usually involves swelling of the affected extremity due to impedance of venous return. Head and neck swelling can occur in advanced SVC obstruction. Presence of superficial venous engorgement can also be present in long-standing obstruction. In acute obstruction due to DVT or complete catheter-associated obstruction in the immediate post-procedural period, venous ischemia can occur. Findings may range in severity from swelling of the extremity and diminished pulses or prolonged capillary refill time, coolness and blue limb discoloration to frank venous ischemia in the threatened limb, classically associated with the physical exam finding of phlegmasia cerulea dolens (Fig. 1). Adolescent patients with chronic lower extremity DVT may present with other symptoms of venous insufficiency including a sense of heaviness, aching pain, and fatigue with activity. These symptoms are collectively described as post-thrombotic syndrome and can be quantitatively described by using the Villalta scoring system (Wik et al. 2017).

Common vessel systems involved include the femoropopliteal and iliac distribution, SVC and innominate veins, and IVC (Frazer and Ing 2009; Tzifa et al. 2007). Patients with unrecognized IVC interruption and azygous continuation may be at high risk for developing IVC thrombosis when there is lower extremity venous catheterization. Malpositioned central venous catheters in the innominate or iliac veins and subsequent long-term delivery of caustic medication, such as parenteral nutrition formulations, contribute to vessel stenosis and occlusion. In May-Thurner syndrome, often affecting adolescent females, there is symptomatic compression and sometimes thrombotic occlusion of the left common iliac vein by the crossing right common iliac artery.

Non-invasive imaging evaluation of SVO begins with Doppler ultrasound (US) examination, which can often be performed in the awake patient. For peripheral disease, most children and adolescents can be adequately evaluated for thrombotic occlusion of major vessels, absence of major conducting pathways, presence of large aberrant channels, and venous reflux. Evaluation of the SVC and IVC is often limited. In the post-intervention setting, Doppler US is used to monitor patients for maintenance of vessel patency following recanalization. Extensive collateral networks are usually not well-evaluated with Doppler US because of the limited field of view of this type of examination.

Contrast-enhanced computed tomography (CECT) is obtained to evaluate central distributions of SVO that are not well-imaged with US, such as in the SVC or upper IVC, as well as to evaluate for atretic or scarred channels and map collateral pathways. Concomitant evaluation for pulmonary embolism can also been obtained with CECT pulmonary angiography in cases where there is concern for DVT. In May-Thurner syndrome, CECT can delineate compression of the left common iliac vein by the crossing right common iliac artery as well as provide for vessel measurement and other aspects of pre-intervention planning. In cases where stent placement is indicated, cross-sectional imaging offers the ability to estimate vessel length and diameter, though caution should be undertaken as underfilling of a vessel upstream from an occlusion may falsely underestimate its true size. Post-stent insertion, CECT can delineate stent patency, intra-stent stenosis, and configuration. Increasingly, cone beam CT (CBCT) is used intra-procedurally to assess for stent apposition or evaluate for the presence of procedural complications.

**Fig. 2:**
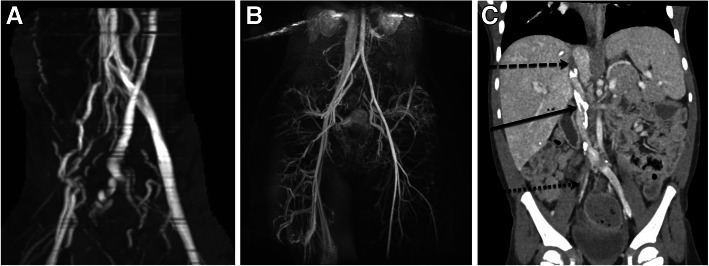
**a** MRV of an 18 y/o M with chronic right lower extremity swelling and DVT demonstrates numerous venous collaterals but no central draining vein beyond the level of the iliac veins. This delayed, post-contrast MIP image shows the numerous venous collaterals in the pelvis and along the spine. **b** MRV of a 16 y/o F with acute onset of left lower extremity DVT shows patency of the right iliac veins draining into the IVC but no opacification of the left iliac veins. The overriding right common iliac artery compresses the left common iliac vein leading to the delayed venous drainage of the extremity and eventual thrombus formation. **c** CT of a 4 y/o M with chronic occlusion of the IVC (dashed arrow), multifocal calcified thrombi in the IVC (solid arrow), and fresh acute clot in the right common iliac vein (dotted arrow). A CT exam in this child allowed for an accurate, high resolution exam for deep venous recanalization planning without having to put the child under general anesthesia to obtain the images

Contrast-enhanced magnetic resonance imaging (MRI) and dynamic MR venography (MRV) is useful for lower extremity and abdominal/pelvic evaluation, especially in patients with long standing disease from congenital anatomic alteration (Fig. 2a) or complex venous disease such as that which is seen in vascular syndromes such as Klippel-Trenaunay syndrome. MRV is the often the exam of choice for non-invasive evaluation of pediatric venous disease due to the lack of ionizing radiation (Fig. 2b); however, the lower resolution of MRI (standard resolution for MRI images is 1-2 mm) must be weighed against the improved resolution of CECT (standard resolution for CT is 0.2–0.3 mm). While in adults this may not seem critical, in children the ability to accurately assess the small caliber of vessels and vast proliferation of venous collaterals often requires the higher resolution available with CECT despite the added radiation dose (Fig. 2c). In patients who have had stent placement, MR evaluation is limited due to the presence of metal artifact. Additionally, MRI in children has the drawback of often requiring general anesthesia for prolonged image acquisition time.

## Procedural equipment and technique

There are basic technical principles that are fundamental to recanalization of any SVO in pediatric or adult patients. Review of all available cross-sectional imaging should be performed to plan for recanalization. Degree of stenosis, presence of collateral pathways, venous thrombosis, and vessel size are examples of considerations that should be evaluated prior to recanalization/stenting. Long-segment or chronic occlusions with obliteration of anatomic pathways pose a risk of procedural failure as well as increased risk of complications from aggressive attempts at recanalization.

First, a high quality digital subtraction angiogram (DSA) should be performed of the region of interest which is free from motion and other artifact. An assessment of whether the occlusion is congenital or acquired, partial or complete, and the presence of collateral pathways should be performed as these considerations will determine the likelihood of success and the degree of aggressiveness that may be undertaken. Once the DSA has been performed, subtraction roadmap overlay is often used to guide catheter and wire passage centrally. Initial attempts at recanalization are often achieved with an angled, hydrophilic, diagnostic catheter (e.g. Kumpe or Berenstein, Cook Bloomington, IN) placed over a hydrophilic 0.018 or 0.035 in. wire. Careful attention should be paid to extraluminal wire perforation.

Planning of access points during recanalization should include consideration of whether access on either side of the occlusion is necessary. For central occlusions, such as in the SVC or IVC, access from both above and below may be necessary to achieve stable positioning for devices. After the secondary access has been obtained, an intravascular snare is used to grasp the floppy end of the wire and pull it out through the primary access, resulting in “flossed” access. When placing a sheath, it may be helpful to nest a small sheath within the larger access sheath to achieve more stability in the region of interest. Sheath size should be selected according to the size of balloon or stent that is required.

In addition to DSA imaging, adjunctive intraprocedural imaging modalities include intravascular ultrasound (IVUS) imaging to better characterize vessel diameter and check for stent apposition, and noncontrast or contrast-enhanced cone beam computed tomography (CBCT). Modern angiographic systems also allow for needle targeting using CBCT navigational overlay upon conventional fluoroscopy, which can be employed to achieve difficult access into target vessels for recanalization.

General principles of venous recanalization are mentioned below with greater detail in the subsequent sections. For acute or short-segment venous stenosis or occlusion, balloon angioplasty followed by maintaining the patient on systemic antiplatelet therapy or anticoagulation may be sufficient for maintenance of patency. However, for chronic or long segment occlusions, stent placement is usually indicated. Pharmacomechanical thrombolysis or thrombectomy should be performed in acute thrombotic SVO (Gaballah et al. 2016).

## Recanalization of acute venous occlusions

Acute venous occlusions (i.e. duration of symptoms less than 14 days) are typically related to acute thromboembolism and etiologies of acute venous occlusions have been well described in numerous publications (Andrew et al. 1994; Menéndez et al. 2016; Gaballah et al. 2016). Thus, this review will focus on procedural technique to cross acute thrombus and subsequent patient management. Typically, acute thrombus burden can be crossed quite easily with a wire as the thrombus has not calcified nor completely hardened. Once wire access is achieved, the method for thrombectomy is selected and may include suction thrombectomy, pharmacomechanical thrombectomy, balloon maceration, or any combination of the above mentioned techniques. Intravascular ultrasound (IVUS) enables the operator to determine if there is an anatomic compression (thoracic outlet syndrome or May Thurner syndrome) and whether the thrombus has completely resolved. Balloon angioplasty is often warranted if there is an area of stenosis; however, stent placement or surgical decompression must be considered in appropriate clinical scenarios (older adolescent patients who may have difficulty clearing the residual thrombus without relief of the chronic venous compression). Patients must be have a carefully constructed plan for anticoagulation, post procedure imaging, and clinic follow up with the Hematology and Interventional Radiology group. If the patient begins to experience recurrent symptoms of venous occlusion, the patient will require either an US, MRI, CECT, or venogram. If the patient remains asymptomatic, follow up imaging can include US, MRI or CECT; an invasive procedure is less likely to be needed.

## Recanalization of chronic venous occlusions

Treatment options for chronic venous occlusions (symptoms for more than 14 days or imaging evidence of chronic thrombus changes such as intraluminal calcifications and prominent venous collaterals) range from surgical bypass grafts to prosthetic graft reconstruction to endovascular angioplasty and stenting (McDevitt et al. 2019). Endovascular treatment is generally considered the first-line therapy in adults and is becoming more widely accepted in pediatrics (Rizvi et al. 2008). Endovascular techniques limit the amount of blood loss, allow for multiple access sites to be re-established during a single procedure, and have favorable long-term patency rates (Sullivan et al. 2018). The importance of pre-procedure planning and high quality pre-procedure imaging cannot be overstated when planning a venous reconstruction. Additionally, even less complex cases of line removals in patients with limited central venous access, should be performed with an intent to preserve and clearly document the current state of SVO with dedicated venograms (Sullivan et al. 2018; Gnannt et al. 2019). Venous access for venous recanalization often requires at least 3 sites of access (range 2–4) and may include lower extremity (femoral or popliteal), upper extremity (basilic, cephalic or brachial), and/or internal jugular venous access (McDevitt et al. 2019).

**Fig. 3:**
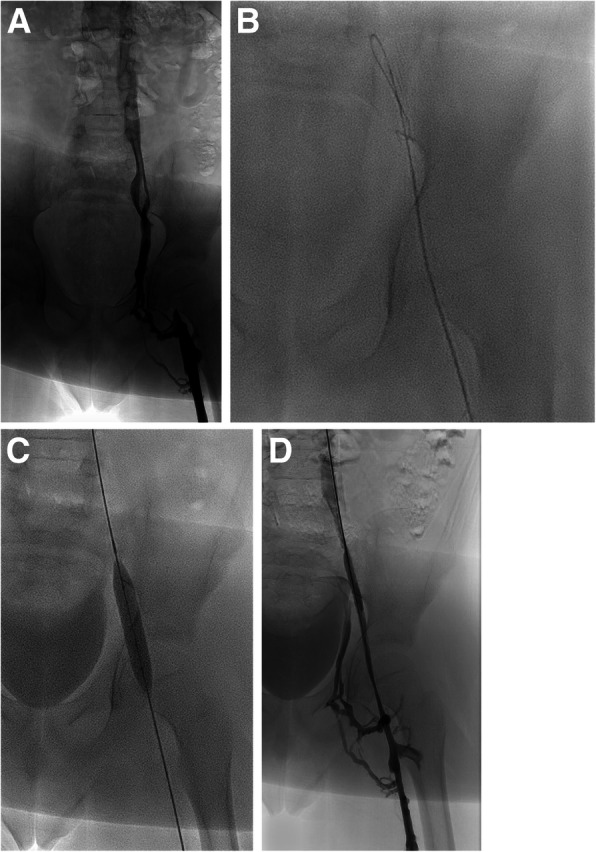
**a** 8 y/o M with history of left lower extremity DVT 1 month after ECMO cannulation for respiratory and cardiac failure. The left femoral vein was used for venous access for ECMO cannula. Venogram demonstrates short segment occlusion of the left external iliac with patent left common femoral, common iliac vein, and IVC. **b** A 0.035″ stiff hydrophilic wire was passed across the occlusion and through the native vessel lumen. **c** Balloon angioplasty was performed with noncompliant balloon; no appreciable waist present with balloon inflation. **d** Final venogram demonstrates a patent channel with persistent collateral vessels and no extravasation or vessel injury

**Fig. 4:**
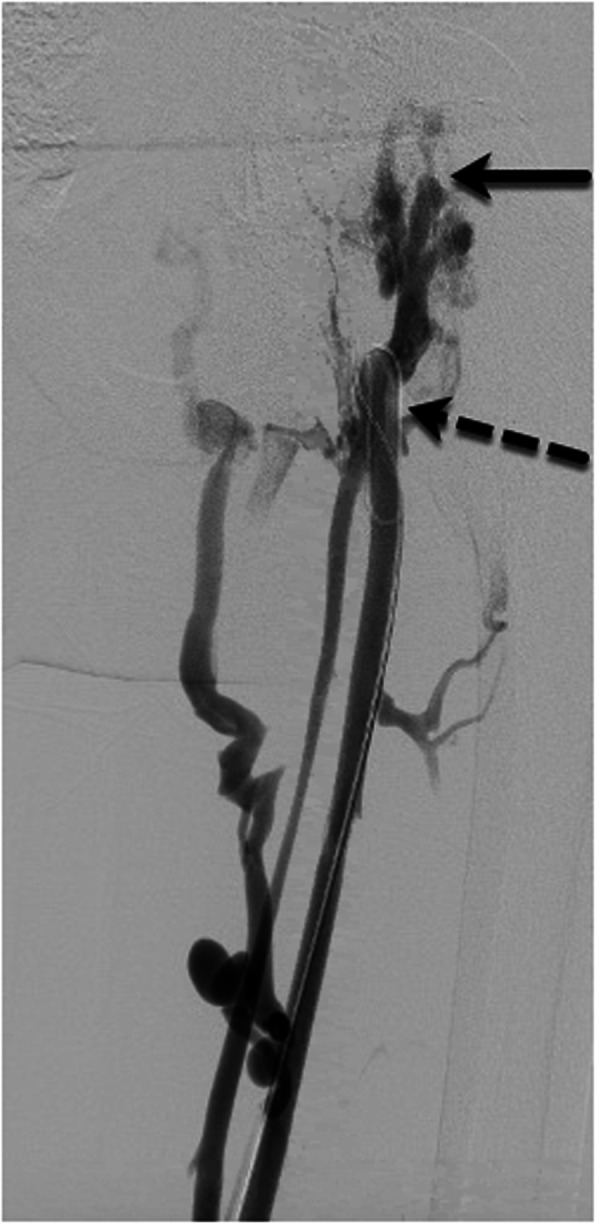
14 y/o M with chronic venous occlusion of the right lower extremity. Solid arrow marks the venous cap: the antegrade entrance to the occluded vessel lumen. Dashed arrow marks the wire looped within the central lumen of the patent portion of the peripheral vessel

**Fig. 5:**
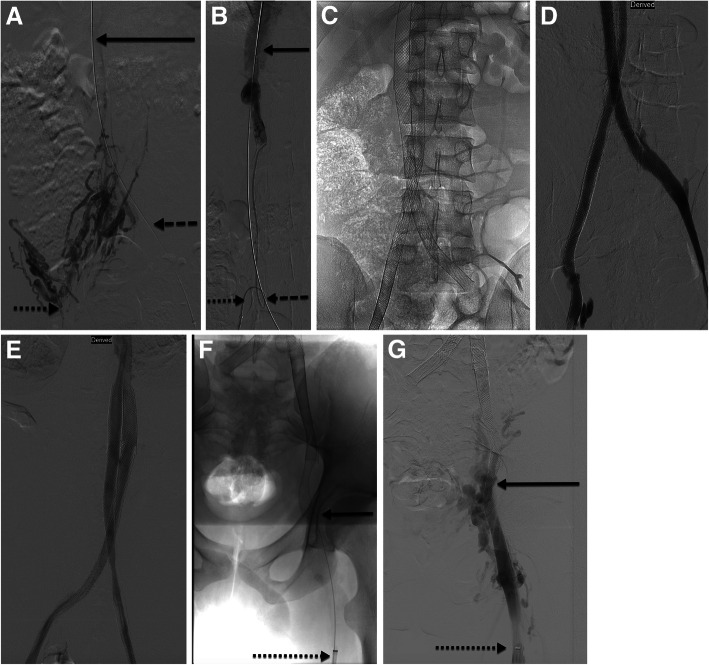
18 y/o M with chronic right lower extremity swelling and DVT presents for planning venogram after MRI demonstrated IVC agenesis from the level of the external iliac to the suprarenal IVC. **a** Initial venogram from the right groin shows numerous diminutive collateral vessels (dotted arrow). A wire was able to be passed from the left groin through collateral vessels (dashed arrow) to the suprarenal IVC (solid arrow). **b** Wires and catheters were eventually able to be passed through retroperitoneal collaterals until the bilateral accesses reached the suprarenal IVC (right groin access marked by dotted arrow, left groin access marked by dashed arrow, IVC solid arrow). **c** IVC reconstruction with stent complex placed from bilateral groins to the suprarenal IVC. **d** Final venogram through stent complex showed excellent outflow with no intrastent stenosis or delay in transit of contrast. **e** Follow up venogram at POD 45, demonstrated continued excellent outflow with no intrastent stenosis or occlusion. Patient’s symptoms of right lower extremity swelling was completely resolved. **f** Follow up venogram 5 months following stent placement, patient had begun to have difficulty with anticoagulation compliance and exhibited symptoms of leg swelling. Unable to pass a wire or catheter beyond the area marked with the solid arrow. **g** Accompanying venogram depicting complete occlusion of the right stent complex with chronic thrombus and numerous collateral vessels draining the right lower extremity (Note, patient is positioned prone). On IVUS, there was kinking of the stent complex near the femoral head which likely was at least partially contributing to the stent complex failure

A central tenet of venous reconstruction recommends operators work from a “normal” beginning point peripherally to a “normal” ending point centrally (van Vuuren et al. 2018). Blunt recanalization techniques involve crossing the occluded vessel length with a hydrophilic, stiff wire and using either the front or back end of the wire to gain purchase centrally (Fig. 3). Vessel size and the chronicity of the occlusion guide the wire choice for recanalization. Microwires used for treating chronic coronary occlusions (examples: 0.014″ Whisper wire, Abbott Vascular Santa Clara, CA and 0.014″ Asahi Confianza, Asahi-Intecc Tustin, CA) have been successful in crossing long lengths of occluded veins with virtually no traumatic insult in the author’s experience (Lawson and Seckeler 2018). While the wire purchase is maintained as centrally as possible, a microcatheter, diagnostic hydrophilic catheter, or small bore sheath is then advanced over the wire. This type of technique is particularly useful in pediatric patients and peripheral venous occlusions. Blunt recanalization can be successful and may make it easier to recanalize the venous outflow through the true lumen of the vessel with reported rates of technical success in the lower extremities and pelvis of up to 86% (McDevitt et al. 2019; Lawson and Seckeler 2018). A peak or cap is often present at the peripheral border of the venous occlusion, marking the antegrade path into the native, occluded vessel. If a venous cap is present on the planning venogram (Fig. 4), efforts for recanalization should be focused on that precise location. If central venous wire access is achieved through collateral vessels in cases of central venous agenesis, the decision to reconstruct through the collateral pathways needs to be carefully considered (Fig. 5). The long term patency rates of long length stent complexes are low, but an emergency situation such as extensive thrombus burden in the lower extremities or severe post thrombotic syndrome may warrant total reconstruction of the IVC in order to provide venous outflow for clearance of thrombus or resolve severe symptoms.

**Fig. 6:**
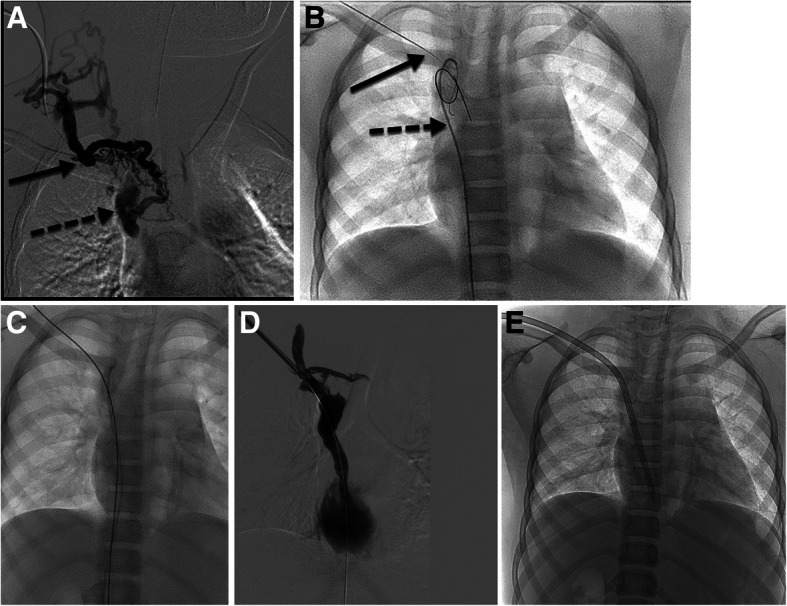
5 y/o M with end stage renal disease requiring hemodialysis with very limited central venous access (left internal jugular occluded and left femoral vein occluded). His hemodialysis catheter was inadvertently pulled out and central venous access lost. **a** Initial venogram from the right neck demonstrates numerous cervical collateral vessels (solid arrow) with no opacification of the right internal jugular vein. Collateral vessels eventually drain into the patent mid SVC and azygous vein (dashed arrow). **b** 22G 10cm Chiba needle passed behind the clavicle (solid arrow) part way with ultrasound guidance and residual guidance with fluoroscopy. Patent SVC target is marked with angled catheter and wire (dashed arrow). **c** Wire is then passed from the neck to the IVC and safety wire from the groin is retracted. Eventually the wire from the neck was snared and “flossed” access was achieved. **d** Post recanalization venogram demonstrates patent but slightly narrowed SVC with limited cervical collateral filling. **e** Hemodialysis catheter placement with tip terminating in the right atrium and tunnel created over the shoulder to reduce the risk of inadvertent catheter removal in the future

**Fig. 7:**
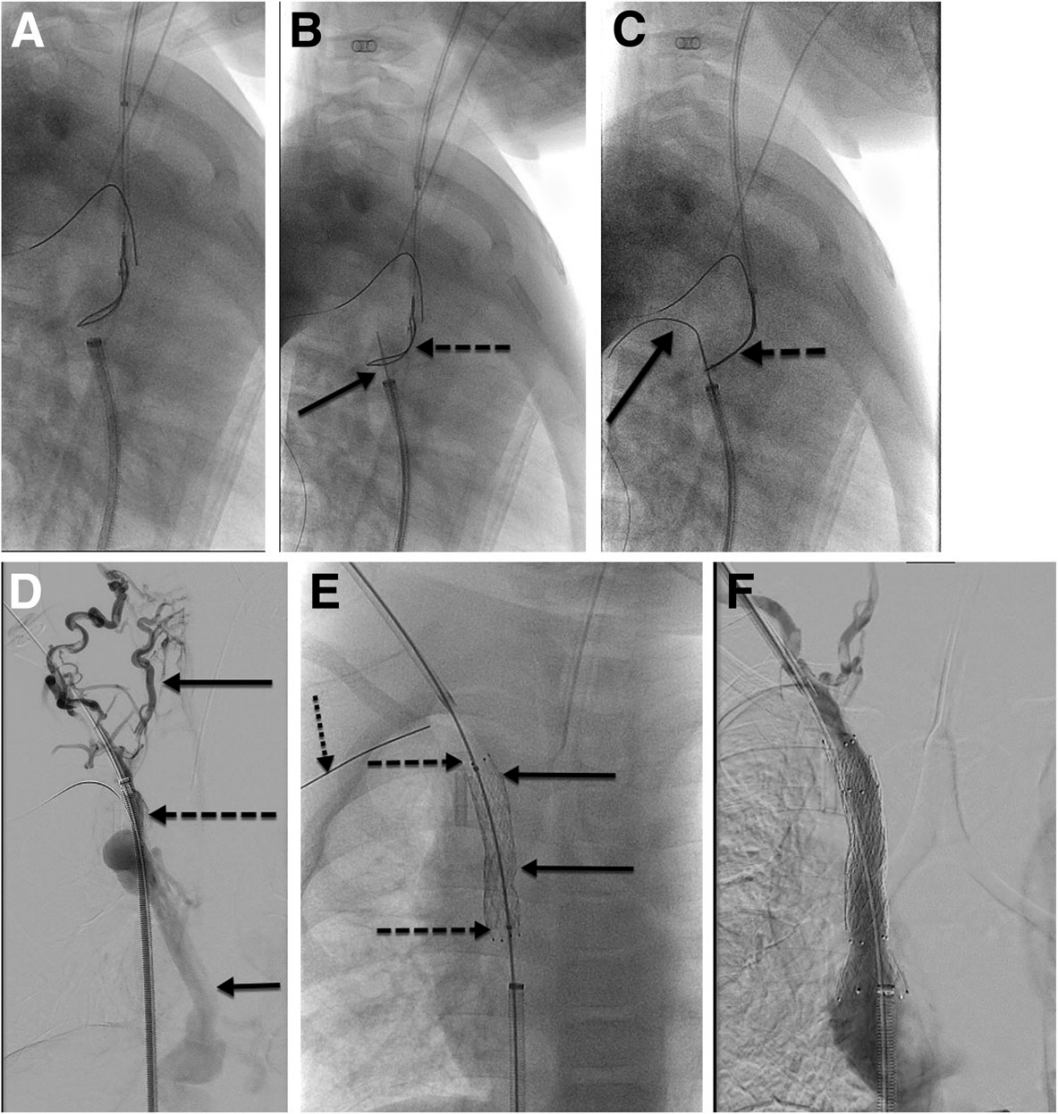
4 y/o M with end stage renal disease requiring hemodialysis access and complete occlusion of the bilateral internal jugular veins and bilateral brachiocephalic veins. Hemodialysis access was from the right groin but he developed thrombus in that vessel and there was concern he would lose his ability for transplant consideration; thus access from the neck was desired. **a** Wire and sheath access from the right groin into the patent portion of the SVC. Snare and sheath access from a collateral vessel in the neck. Wire is also noted on the left periphery of the image marking the subclavian vein into the azygous vein. **b** A 22G 65 cm Chiba needle (solid arrow) was passed from the right groin sheath into the snare (dashed arrow) from the right neck venous access. **c** Inner obturator of needle was removed and replaced with a 0.018″ hydrophilic wire. Wire (solid arrow) was advanced centrally and snare tightened on wire (dashed arrow) while needle was retracted. **d** Sheath was advanced through the occlusion (dashed arrow) and venogram performed. Demonstrated numerous collateral venous vessels (upper solid arrow) with patent azygous vein (lower solid arrow) but no opacification of the occluded SVC. **e** Stent complex: balloon mounted Palmaz stent (Cordis Baar, Switzerland) deployed at the level of the complete occlusion (two solid arrows marking the cranial and caudal extent of the stent) and a self-expanding, Zilver 14 mm stent (Cook Bloomington, IN) deployed within the Palmaz stent. The stent complex connected the patent vessel lumens (two dashed arrows marking the cranial and caudal extent of the stent). Center of stent complex only ballooned to 10 mm due to size of patient native vasculature with intent of subsequent procedures to increase stent diameter as the patient grows. Dotted arrow marks the wire in the subclavian vein. **f** Final venogram status post recanalization, stenting, and balloon angioplasty. Contrast readily fills the right atrium with no extravasation present

Sharp recanalization usually is performed with small gauge, non-coring needles under US, fluoroscopy, or CBCT guidance. The needle length can be variable depending on patient size and distance from the access site to the occlusion (7 cm, 10 cm, 15 cm, or 65 cm). The planning venogram must be scrutinized for the best approach and direction to cross the occlusion: the length of occlusion, the trajectory, and any at risk adjacent structures while crossing the occluded portion of the vessel (lung, pericardium, bowel, etc). The needle can be guided through a direct percutaneous approach (through the skin into the patent vessel and across the occlusion) or via a catheter coaxially loaded into a vascular sheath (sheath into patent vein, catheter advanced to the site of occlusion, and needle through the catheter and eventually across the occlusion). If the direct percutaneous route is chosen for sharp recanalization, a 0.018″ or 0.014″ wire is directly passed into the central, patent vein (Fig. 6). The percutaneous tract is then serially dilated until a sheath is able to be passed over a wire and the rest of the procedure performed. If the coaxial system is chosen for sharp recanalization, the needle is coaxially loaded through an angled catheter, directed towards the occlusion, and passed across (Fig. 7). Typically, a loop snare is placed in the target vessel and once the needle is passed into the snare, the snare is tightened over the needle. The inner obturator is removed from the needle, a 0.018″ wire is passed through the needle, and the snare is then tightened around the wire. The wire is pulled into the snare catheter thereby establishing “flossed” access across the occlusion. A catheter or long tapered sheath is advanced across the wire through the occlusion, and then the wire can be exchanged to a larger, stiffer wire (Amplatz, Rosen, or Lunderquist Cook Bloomington, IN) if necessary.

Sharp recanalizations of venous occlusions are often successful, but also carry significant risk if the anticipated needle pass cannot avoid crossing through critical structures or the occlusion length is too long (usually more than 3 cm). The use of intravascular ultrasound (IVUS) has lessened the risk of sharp venous recanalizations. The IVUS-assisted technique for chronic venous occlusions was adapted from the well-described use of IVUS in transjugular intrahepatic portosystemic shunt (TIPS) creation (Farsad et al. 2012; Kao et al. 2016). A longitudinal, side-firing IVUS is placed in the central target vein instead of a snare device, and the complementary US system is used to display the visualized vascular structures. The active tip of the IVUS probe is angled until the needle tip is visualized, which allows for real time visualization of the needle pass in relation to the surrounding structures. Once the needle is confirmed to be within the lumen of the target vein, a wire is advanced centrally. The IVUS probe is removed and exchanged for a snare device; the wire is snared and “flossed access” is achieved across the occlusion. Real time visualization allows for path correction as well as making fewer passes to achieve the recanalization.

There are various other endovascular techniques described in the literature to cross refractory chronic venous occlusions; rates of such refractory occlusions are reported to be as high as 18% in adult literature (Keller et al. 2018). One such technique is radiofrequency wire-aided traversal. The radiofrequency wire (RF) is more commonly used in adults than pediatrics; however, the technique may be applicable to adolescent/near-adult sized patients and should be discussed for thoroughness in this review article. RF wires have been reported in the literature for small cohort studies and retrospective reviews (total number of cases is approximately less than 90 cases) (Keller et al. 2018). The RF wire is used to traverse occlusions in the SVC, brachiocephalic, IVC, and iliac veins with multiple sites of venous access on either side of the occlusion for the procedure. The RF wire is often used only after more standard techniques to cross the occlusion have failed (multiple combinations of hydrophilic stiff wires, catheters and sheaths). The RF wire is positioned on one side of the occlusion with a target snare on the opposite side of the occlusion; the RF wire was advanced slowly under fluoroscopy and/or cone beam CT. When the RF wire makes contact with the metal snare, the system is short circuited which serves as the final confirmation the occlusion had been crossed. In a retrospective review, the mean length of occlusion crossed by the RF wire was 10.05 cm (range 0.8–31.7 cm, median 5.2 cm) (Keller et al. 2018). Technical success rates are reported as high as 80% and 91% (Keller et al. 2018; Sivananthan et al. 2015); and while complication rates are low (roughly 2–5%), the inadvertent injuries tend to be major complications such as tracheal or SVC perforation. Given a favorable anatomic location and carefully selected patient, RF wires should remain a consideration for those who are comfortable and experienced in using them in a refractory chronic venous occlusion.

Once the access is achieved across the occlusion, the patient should be heparinized as the following steps of angioplasty and possible stenting lead to a prothrombotic milieu. At the authors’ institution, the typical loading dose of heparin is 100 units/kg up to max dose of 5000 units. With the wire access established, balloon angioplasty is next step to restoring patency. Noncompliant balloons such as Conquest (BD Bard Tempe, AZ), Dorado (BD Bard Tempe, AZ), Atlas (BD Bard Tempe, AZ), and Powerflex (Cordis Santa Clara, CA) are often used; sizing is based on venograms and IVUS measurements of the vessels flanking the occlusion.

Once a patent channel has been established, stenting is usually performed if the child is skeletally mature and the location of the proposed stents is conducive to stenting. If the child is not skeletally mature, stents can still be considered in the supradiaphragmatic region (i.e. brachiocephalic and SVC) as long as the stent complex can be serially ballooned to a larger caliber over time. This is often achieved with placing a self-expanding stent through a balloon mounted stent; the overall stent diameter is thereby constrained by the smaller, balloon-expandable stent. The “hourglass” shaped, constrained stent complex will provide a controlled expansion with follow up angioplasty procedures to expand the total stent diameter as the child grows (Image 7E). If the location of the occlusion is not amenable to stent placement (near a joint or in the extremity) or if the child is not skeletally mature, repeat balloon angioplasty, potentially with drug eluting balloons, and prophylactic anticoagulation is considered the safest treatment (Image 3D).

SVO which typically require stent placement for long term patency include SVC, IVC, and iliac veins (May Thurner). Common stents used in these locations include Wallstent (Boston Scientific Marlborough, MA), Palmaz (Cordis Baar, Switzerland), Viabahn (Gore Medical Flagstaff, AZ), S.M.A.R.T. Control stent (Cordis Baar, Switzerland), and the recently introduced Venovo (BD Bard Tempe, AZ) and Vici stent (Boston Scientific Marlborough, MA). Only in select circumstances, stents are deployed below the inguinal ligament. Van Vuuren et al. described experience in stenting into the common femoral vein in 79 adult patients. Fourteen of the 79 patients had primary stent placement for post-thrombotic changes that extended peripherally to the femoral confluence, but the deep femoral vein had to provide the majority of venous inflow in order to maintain stent patency (van Vuuren et al. 2018).

Following delivery of the stent, angioplasty is performed to ensure the stent is adequately dilated and the stent wall abuts the vessel wall. Final diameter of the stent is determined based on the prior venograms and IVUS measurements (Haddad et al. 2018). Post stent venogram should show decreased number of adjacent collaterals, in-line flow of the deep venous structures, and no filling defects within the stent complex. There is a well-described phenomenon of apparent narrowing in the external iliac vein after placement of an adjacent common iliac venous stent and is thought to represent an imaging phenomenon with no clear clinical significance (Al-Hakim et al. 2018). IVUS is also helpful in evaluating the stent apposition, diameter, and coverage across the occlusion. Areas of slight intrastent stenosis or small, residual thrombus are easily identified with IVUS and may be missed with venography.

Anticoagulation or antiplatelet therapy should be initiated as soon as the stent complex is in place. After consultation with the Hematology service, the best agent is selected and prescribed for the patient. Despite the importance of maintaining stent patency in venous recanalizations, the literature does not suggest on particular method or agent over another. Various options include low molecular weight heparin (LMWH) with dual anti-platelet agents (enoxaparin, clopidogrel, and aspirin) where clopidogrel was only used for 2 months following stent placement (McDevitt et al. 2019), dual antiplatelet agents (clopidogrel and aspirin) (Lawson and Seckeler 2018), and LMWH and aspirin (Sullivan et al. 2018). Little to no data exists for anticoagulation with direct oral anticoagulants in children with chronic central venous occlusion relieved by stent deployment.

Follow up imaging after recanalization is imperative for long term management. In pediatrics, US or MRI is often preferred over CECT. However, US may under or overestimate stent patency due to operator error and MRI may be unable to answer the question of stent patency or stenosis due to metal artifact disturbances. Thus, if there is considerable concern whether the stent remains patent or not, it is often best answered with a diagnostic venogram or CECT. If IVUS is used during the diagnostic venogram, contrast and radiation doses can further be lowered. Primary patency rates of systemic venous occlusion treated with angioplasty alone are not well reported; data is just beginning to emerge for drug eluting balloon angioplasty in venous stenosis/occlusion and long term patency rates for this therapy have not been corroborated in pediatric cohorts. Primary patency rates for children with SVC syndrome (non-malignant occlusion) and stent placement are not well reported in the literature as there are predominantly case reports with no translatable data for larger patient groups. Primary patency rates in pediatric patients with IVC and iliac vein stents range from 64 to 100% in the first 12 months following stent placement (McDevitt et al. 2019; Sullivan et al. 2018; Lungren et al. 2018). Primary assisted and secondary patency rates were 100% in all studies (McDevitt et al. 2019; Lungren et al. 2018). Primary patency of left common iliac venous stents placed in adolescents for May Thurner have been reported at 79% and 89% for secondary patency (Lungren et al. 2018). However, rates of stent patency are not reliably reported beyond 36 months in most studies whether it is due to loss of patients to follow up or disparate mechanisms for documenting further interventions needed to maintain stent patency. The poor data rates for stent patency is particularly concerning when considering the life of the stent in a pediatric patient. Additional studies to better analyze existing data and propose solutions to routine follow up for stent maintenance are needed.

## Complications from venous recanalization

The most common complication during recanalization is establishing an extraluminal tract from the desired vessel lumen. While this is usually of low consequence due to the low pressure venous system, it can become higher risk depending on the size of puncture, size of vessel perforated (IVC rent is more detrimental than a common femoral vein rent), and any adjacent tissue damage. Plans to immediately respond to this type of complication should be made and resuscitation equipment readily available if the need arises. Typically, an angioplasty balloon can be inflated for a prolonged time (3 min of sustained inflation) across the vessel injury with subsequent venogram and/or IVUS to prove there is no further extravasation (McDevitt et al. 2019). If the balloon insufflation does not seal the injury, a covered stent can be deployed across the injury to permanently resolve the injury. Covered stenting is necessary when the vessel wall or adjacent pericardium has been torn from either angioplasty or stenting of the SVC, and the pericardial space fills with blood which may lead to cardiac tamponade. In some instances, a pericardial drain may also be required to decrease the pressure on the heart (Haddad et al. 2018).

The majority of other complications are related to thromboembolic events: embolization of a thrombus, intrastent thrombosis, or recurrent thrombosis in a newly recanalized vessel. Due to the amount of disruptions to the native endothelium, it is imperative the patient is anticoagulated during and after the procedure. If the thromboembolic event causes impaired venous drainage of the limb or hemodynamic instability, suction thrombectomy, balloon maceration, or catheter directed thrombolysis may be warranted. Risks and benefits of each therapy should be considered given the overall health status of the patient (i.e. contraindications for receiving tissue plasminogen activator).

## Conclusion

In summary, SVO in pediatrics is increasing in incidence due to better detection of congenital occurrences as well as increased utilization of central venous devices and venous thromboembolic disease. Interventional radiology is often consulted for difficult central venous access cases and must be ready to suggest a life-saving option such as venous recanalization when feasible. Acute thrombotic occlusions are typically treated with pharmacomechanical thrombolysis, angioplasty, and possible stent placement and if aggressively treated, may allow the child to have a lifelong course of vessel patency. Chronic venous occlusions can prove to be a more challenging problem but attempts at recanalization through various endovascular techniques and maneuvers can be beneficial. The data regarding stent patency is limited at best in the pediatric world and further studies better delineating the anticoagulation regimens and principles for long term stent management are very much needed.

## Data Availability

Data sharing is not applicable to this article as no datasets were generated or analysed during the current study.

## Abbreviations

SVO: Systemic venous occlusion
IVC: Inferior vena cava
SVC: Superior vena cava
DVT: Deep venous thrombosis
US: Ultrasound
CECT: Contrast-enhanced computed tomography
MRI: Magnetic resonance imaging
MRV: Magnetic resonance venography
DSA: Digital subtraction angiogram
CBCT: Cone beam computed tomography
IVUS: Intravascular ultrasound
RF: Radiofrequency

## References

[CR1] Al-Hakim RA, Kaufman JA, Farsad K (2018). Iliac vein stent placement: acute Venographic changes and relevance to venous biomechanics. J Vasc Interv Radiol.

[CR2] Andrew M, David M, Adams M (1994). Venous thromboembolic complications (VTE) in children: first analyses of the Canadian registry of VTE. Blood.

[CR3] Bass JE, Redwine MD, Kramer LA (2000). Spectrum of Congenital Anomalies of the Inferior Vena Cava: Cross-sectional Imaging Findings: (CME available in print version and on RSNA Link). RadioGraphics.

[CR4] Farsad K, Fuss C, Kolbeck KJ (2012). Transjugular intrahepatic Portosystemic shunt creation using intravascular ultrasound guidance. J Vasc Interv Radiol.

[CR5] Frazer JR, Ing FF (2009). Stenting of stenotic or occluded iliofemoral veins, superior and inferior vena cavae in children with congenital heart disease: acute results and intermediate follow up. Catheter Cardiovasc Interv.

[CR6] Gaballah M, Shi J, Kukreja K (2016). Endovascular thrombolysis in the Management of Iliofemoral Thrombosis in children: a multi-institutional experience. J Vasc Interv Radiol.

[CR7] Gnannt R, Chamlati R, Waespe N (2019). Clinical impact of chronic venous changes induced by central lines in children: a cohort with abnormal Venograms. J Vasc Interv Radiol.

[CR8] Haddad MM, Thompson SM, McPhail IR (2018). Is long-term anticoagulation required after stent placement for benign superior vena cava syndrome?. J Vasc Interv Radiol.

[CR9] Kao SD, Morshedi MM, Narsinh KH (2016). Intravascular ultrasound in the creation of Transhepatic Portosystemic shunts reduces needle passes, radiation dose, and procedure time: a retrospective study of a single-institution experience. J Vasc Interv Radiol.

[CR10] Keller EJ, Gupta SA, Bondarev S (2018). Single-center retrospective review of radiofrequency wire recanalization of refractory central venous occlusions. J Vasc Interv Radiol.

[CR11] Lawson EN, Seckeler MD (2018) Successful percutaneous recanalization of a chronically occluded inferior vena cava in a young child. World J Pediatr Congenit Heart Surg:215013511877131. 10.1177/2150135118771316 10.1177/2150135118771316 30296929

[CR12] Lungren MP, Towbin AJ, Roebuck DJ (2018). Role of interventional radiology in managing pediatric liver tumors: part 1: endovascular interventions. Pediatr Radiol.

[CR13] McDevitt JL, Srinivasa RN, Hage AN (2019). Lower extremity endovenous reconstruction for symptomatic occlusive disease in pediatric patients: techniques, clinical outcomes, and long-term stent patencies. Pediatr Radiol.

[CR14] Menéndez JJ, Verdú C, Calderón B (2016). Incidence and risk factors of superficial and deep vein thrombosis associated with peripherally inserted central catheters in children. J Thromb Haemost.

[CR15] Rizvi AZ, Kalra M, Bjarnason H (2008). Benign superior vena cava syndrome: stenting is now the first line of treatment. J Vasc Surg.

[CR16] Sivananthan G, MacArthur D, Daly K et al Safety and efficacy of radiofrequency wire recanalization of chronic central venous occlusions. J Vasc Access 16:309–314. 10.5301/jva.5000360 10.5301/jva.5000360 25656250

[CR17] Sullivan PM, Merritt R, Pelayo JC, Ing FF (2018). Recanalization of occluded central veins in a parenteral nutrition–dependent child with no access. Pediatrics.

[CR18] Tzifa A, Marshall AC, McElhinney DB (2007). Endovascular treatment for superior vena cava occlusion or obstruction in a pediatric and young adult population. J Am Coll Cardiol.

[CR19] van Vuuren T, Wittens C, de Graaf R (2018). Stent extension below the common femoral vein in extensive chronic Iliofemoral venous obstructions. J Vasc Interv Radiol.

[CR20] Wik H, Ghanima W, Sandset P, Kahn S (2017). Scoring Systems for Postthrombotic Syndrome. Semin Thromb Hemost.

